# Association Between Thyroid Gland Volume and Postoperative Hypothyroidism Following Hemithyroidectomy

**DOI:** 10.1002/oto2.70239

**Published:** 2026-04-27

**Authors:** Mayas Arraf, Alaa Safia, Adi Sharabi‐Nov, Moanes Awad, Yaniv Avraham, Ohad Ronen, Shlomo Merchavy

**Affiliations:** ^1^ Department of Otolaryngology Head and Neck Surgery, Ziv Medical Center Safed Israel; ^2^ Statistics Unit, Ziv Medical Center, Safed Israel and Tel‐Hai Academic College, Tel‐Hai Israel; ^3^ Department of Radiology Ziv Medical Center Safed Israel; ^4^ The Azrieli Faculty of Medicine Bar Ilan University Ramat Gan Israel; ^5^ Department of Otolaryngology Head and Neck Surgery, Galilee Medical Center Nahariya Israel

**Keywords:** hemithyroidectomy, hypothyroidism, outcomes, thyroid surgery, thyroid volume

## Abstract

**Objective:**

To evaluate whether the proportion of resected thyroid tissue or the residual lobe volume predicts postoperative hypothyroidism following hemithyroidectomy.

**Study Design:**

Multicenter retrospective cohort study.

**Setting:**

Ziv Medical Center and Galilee Medical Center, Israel.

**Methods:**

The study included 134 adult patients who underwent hemithyroidectomy between 2017 and 2024. Patients with preexisting hypothyroidism or malignant histopathology were excluded. Preoperative thyroid gland volumes were measured by computed tomography (CT) and correlated with serum thyroid hormone profiles (T3, T4, and TSH) obtained preoperatively, and at 1 and 6 months postoperatively. Associations between thyroid volume parameters and postoperative hypothyroidism were analyzed statistically.

**Results:**

Postoperative hypothyroidism developed in 29.1% of patients, within the first postoperative month. Affected patients exhibited significantly higher baseline TSH levels than euthyroid counterparts (3.0 vs 1.4 μIU/mL, *P* < .001). No significant correlation was found between postoperative thyroid function and total gland volume, resected lobe volume, or residual‐to‐total volume ratio. At 6 months, only 12% of patients remained hypothyroid, suggesting partial recovery of thyroid function in the remaining lobe.

**Conclusion:**

Neither the extent of resection nor the residual thyroid volume predicted postoperative hypothyroidism. These findings imply that factors beyond gland size—such as preoperative functional reserve or intrinsic thyroid health—may play a more decisive role in maintaining postoperative endocrine stability. Identifying these predictors could enhance patient selection, risk stratification, and postoperative follow‐up.

Hypothyroidism is the most common complication of hemithyroidectomy for thyroid nodules.^1^, ^2^ Hemithyroidectomy is the preferred surgical procedure for managing benign unilateral thyroid conditions, as it preserves secretion of endogenous thyroid hormones in the remaining lobe and can diminish the necessity of exogenous hormonal supplementation.^2^, ^3^ The incidence of posthemithyroidectomy hypothyroidism is notable, with multiple studies reporting a pooled incidence rate of approximately 29%, encompassing both overt and subclinical forms.^4^ Chotigavanich et al found that 27% of patients developed hypothyroidism postsurgery, with 6% experiencing overt hypothyroidism and 21% subclinical hypothyroidism.^5^

The underlying mechanisms contributing to the development of posthemithyroidectomy hypothyroidism are primarily multifactorial.^6^ Several risk factors for the development of postoperative hypothyroidism after hemithyroidectomy have been reported, including elevated preoperative thyroid‐stimulating hormone (TSH) levels, female sex, positivity for antithyroid peroxidase antibody (TPOAb) and anti‐thyroglobulin antibody (TgAb), advanced age, malignancy, right‐sided hemithyroidectomy and minor residual thyroid tissue observed on ultrasonography.^7^ However, these risk factors vary among studies, and no consensus has been established.

Previous studies have shown that the volume of remnant thyroid left following surgery can be correlated with the risk of developing hypothyroidism; a smaller remnant volume compromises the thyroid gland's ability to produce sufficient hormones.^8^, ^9^ A study by Brian Hung‐Hin Lang et al found that a smaller thyroid remnant volume significantly increases the risk of hypothyroidism after a hemithyroidectomy. A significant inverse association between the preoperative contralateral lobe's volume and hypothyroidism risk was observed after hemithyroidectomy.^10^ Hyeong‐Gon Moon et al have found that patients with a high preoperative TSH level and small remnant thyroid volume are at high risk of developing hypothyroidism following hemithyroidectomy.^11^

Both studies have used ultrasonography for thyroid size evaluation. In this study, we used CT scans to evaluate thyroid lobe size preoperatively to be correlated with T3, T4, and TSH blood levels for thyroid function follow‐up.

Although ultrasonography is considered the standard modality for thyroid volume assessment,^12^ CT was used in the present study because all included patients had preoperative CT imaging available for surgical planning. This allowed standardized, reproducible, and operator‐independent volumetric measurements across both participating centers.

## Methods

### Participants

This retrospective multicenter study included 134 adult patients who underwent hemithyroidectomy between March 2017 and October 2024 at Ziv Medical Center and Galilee Medical Center, Israel.

The study was approved by the Institutional Review Board of Ziv Medical Center (Approval No. 0035‐21‐ZIV). Patients with preoperative hypothyroidism, known preoperative malignant thyroid disease, or age under 18 years were excluded.

### Data Collection

Demographic (age, gender, BMI, and ethnicity) and clinical data (levels of T3, T4, and TSH, Bethesda category, type of surgery, and side of the resected lobe) were collected from patients' electronic medical records.

Preoperative thyroid gland volumes of both lobes were calculated based on computed tomography (CT) scans. All volumetric measurements were performed by a single experienced radiologist who was blinded to postoperative thyroid function outcomes.

Thyroid lobe volume was calculated using 3 orthogonal diameters (anteroposterior, transverse, and craniocaudal), applying the ellipsoid formula (length × width × depth × 0.52). The volume of the resected lobe was derived exclusively from the preoperative CT scan using the same method; no postoperative specimen measurements were used, in order to avoid bias related to postresection tissue deformation or volume change.

The resected lobe ratio was defined as the volume of the resected lobe divided by the total preoperative thyroid volume.

Serum levels of T3, T4, and TSH were collected preoperatively and at 1 and 6 months postoperatively.

Due to the very small number of patients in individual Bethesda subcategories, categories were grouped for descriptive analysis only, and no inferential conclusions were drawn from these distributions.

### Determination of T3, T4, and TSH Levels

Thyroid function was evaluated at several time points: preoperatively, and at 1 and 6 after surgery. Subclinical hypothyroidism was defined, according to the 2012 ATA/AACE guidelines,^13^ as a thyroid‐stimulating hormone (TSH) level of 4.20 to 10 µIU/mL with normal free T4 in asymptomatic patients. Overt hypothyroidism was defined as a TSH level >10 µIU/mL in combination with a reduced free T4 level. For this study, both conditions were collectively classified as “hypothyroidism.”

Thyroid hormone replacement therapy was initiated in patients with TSH > 10 µIU/mL or a low free T4 level, especially when accompanied by clinical symptoms such as fatigue, weight gain, or depression. These management thresholds were based on established clinical guidelines and institutional protocols.

The time to onset of hypothyroidism was defined as the interval between the date of surgery and the first documented biochemical evidence of hypothyroidism. To minimize bias and ensure consistency, 2 independent members of the research team reviewed patient symptoms and laboratory data. Symptom assessment was conducted using structured screening questions during follow‐up visits and supplemented with patient or caregiver reports.

Serum TSH and total thyroxine (T4) levels were measured preoperatively and at 1 and 6 months postoperatively.

The serum levels of T3, T4, and TSH were measured by the electrochemiluminescence immunoassay (ECLIA) method for use on the Cobas e immunoassay analyzer, using the following Roche Diagnostics GmbH. kits Ref. 11731360 for T3, 06437281 for T4 and 08429324 for TSH. For each kit, the normal expected values correspond to the 2.5th and 97.5th percentiles.

### Statistical Analysis

Categorical variables were presented as frequencies (n) and percentages, while continuous variables were presented as medians (Me) and interquartile ranges (IQR) since they were not normally distributed. Differences in continuous variables between the 2 study groups were tested using the Mann‐Whitney non‐parametric test. Relationships between the study groups and categorical variables were tested using Pearson's chi‐squared or Fisher's exact test (depending on the sample size). Analyses were performed using SPSS software, version 29 (IBM), with a statistical significance threshold of *P* < .05.

## Results

Out of 134 patients who underwent lobectomy, 39 (29.1%) developed postoperative hypothyroidism. Patients in the hypothyroidism group were slightly older than those who remained euthyroid, with a median age of 58 years compared to 54 years, although this difference was on the verge of statistical significance (*P* = .083, Table 1).

**Table 1:** Demographic and Clinical Characteristics According to the Development of Hypothyroidism After a Month

	Hypothyroidism		
Characteristics	None (n = 95)	Yes (n = 39)	*P*
Demographic			
Age, years (Me, IQR)	54, 43‐66	58, 52‐68	.083
Gender, female (n, %)	77, 81.1	30, 76.9	.588
Ethnicity (n, %)			
Jewish	43, 453	22, 56.4	.241
Arab	52, 54.7	17, 43.6	
BMI, kg/h^2^ (Me, IQR)	28, 24‐32	29, 26‐31	.396
Clinical			
Diabetes mellitus (n, %)	21, 22.1	5, 12.8	.217
Diagnosis (n, %)			
Goiter	73, 76.8	31, 79.5	.944
PTC	14, 14.7	5, 12.8	
Follicular adenoma	8, 8.5	3, 7.7	
Bethesda (n, %)			
1	1, 1.1	2, 5.1	.147
2‐6	94, 98.9	37, 94.9	
Side of operated lobe (n, %)			
Left	50, 52.6	19, 48.7	.681
Right	45, 47.4	20, 51.3	
Thyroid volume, cc (Me, IQR)	44, 27‐79	46, 27‐74	.719
Resected lobe volume, cc (Me, IQR)	33, 18‐57	32, 21‐57	.587
Resected lobe Ratio, cc (Me, IQR)	0.72, 0.61‐0.85	0.81, 0.66‐0.90	.173

Abbreviations: IQR, interquartile range; Me, median.

As shown in Table 1, most patients in both groups were female (76.9% vs 81.1%, *P* > .05). Ethnic distribution showed a higher proportion of Jewish patients in the hypothyroidism group (56.4% vs 45.3%), whereas Arabs were more common in the euthyroid group (43.6% vs 54.7%), but the difference was not significant (*P* > .05). Median BMI was comparable between groups (29 vs 28 kg/m², *P* = .396). The prevalence of diabetes mellitus was lower in the hypothyroidism group (12.8% vs 22.1%), though this was not statistically significant (*P* > .05).

Concerning clinical and pathological characteristics, the distribution of final diagnoses was similar across groups: goiter was the most common indication for surgery (79.5% vs 76.8%), followed by papillary thyroid carcinoma (12.8% vs 14.7%) and follicular adenoma (7.7% vs 8.5%), with no significant difference (*P* > .05). Although patients with known preoperative malignancy were excluded, a small number of patients were found to have incidental papillary thyroid carcinoma on final histopathology. These cases were retained in the analysis because all had normal preoperative thyroid function, no evidence of autoimmune thyroid disease, and did not require immediate completion thyroidectomy or adjuvant therapy, thereby minimizing the likelihood that their inclusion biased the assessment of postoperative thyroid function outcomes.

Bethesda classification did not differ significantly between groups (*P* = .147). Although category 1 appeared numerically more frequent in the hypothyroidism group (2 vs 1 patient), the absolute numbers were very small and no statistically or clinically meaningful conclusion can be drawn from this finding.

The side of the operated lobe was balanced between groups, with left‐sided resections in 48.7% versus 52.6% and right‐sided resections in 51.3% versus 47.4% (*P* > .05) (Table 1).

Thyroid volumetric parameters were also largely comparable. Median thyroid volume was 46 cc in the hypothyroidism group and 44 cc in the euthyroid group (*P* > .05). Similarly, resected lobe volume was nearly identical between groups (32 cc vs 33 cc, *P* > .05). However, patients who developed hypothyroidism had a slightly higher resected lobe ratio compared to those without hypothyroidism (0.81 vs 0.72), although this trend did not reach statistical significance (*P* > .05) (Table 1).

Table 2 describes the T3, T4 and TSH levels at 3 measurement times. At baseline, none of the patients were hypothyroid. Preoperatively, T3 and T4 levels were comparable between groups, while median TSH was significantly higher in patients who later developed hypothyroidism (3.0 vs 1.4 μIU/mL, *P* < .001). One month after surgery, 29% of patients had developed hypothyroidism, T4 levels were significantly lower in the hypothyroidism group compared to the euthyroid group (13.9 vs 15.0 pmol/L, *P* < .05), while T3 remained similar. TSH was markedly elevated in the hypothyroidism group (5.6 vs 1.8 μIU/mL, *P* < .001).

**Table 2:** T3, T4 and TSH Levels According to Measurement Time (Me, IQR)

		Hypothyroidism			
Time	Thyroid hormone	None (n = 95)	Yes (n = 39)	*P*	Patients with hypothyroidism^a^
Presurgery	T3 (pmol/L)	4.9, 4.2‐5.0	4.7, 4.3‐5.0	.965	
	T4 (pmol/L)	15.0, 14.0‐16.0	14.8, 12.7‐16.0	.072	0%
	TSH (μIU/mL)	1.4, 0.9‐2.1	3.0, 1.7‐3.7	<.001	
A month from surgery	T3 (pmol/L)	4.7, 4.0‐5.0	4.6, 4.1‐5.0	.691	
	T4 (pmol/L)	15.0, 13.8‐16.0	13.9, 11.0‐16.0	.025	29%
	TSH (μIU/mL)	1.8, 1.1‐2.8	5.6, 3.1‐6.5	<.001	
6 months from surgery	T3 (pmol/L)	4.8, 4.2‐5.1	4.5, 4.1‐5.0	.112	
	T4 (pmol/L)	14.9, 14.0‐15.9	14.3, 13.0‐16.0	.139	12%
	TSH (μIU/mL)	1.4, 1.0‐1.9	3.0, 1.3‐4.4	<.001	

Data presented as median and interquartile range.

^a^ According to levels of T4 and TSH (T4 < 12 pmol/L, TSH > 4.2 μIU/mL).

At 6 months postoperatively, 12% of patients remained hypothyroid. T3 and T4 levels did not differ significantly between groups, although TSH remained elevated in those with hypothyroidism (3.0 vs 1.4 μIU/mL, *P* < .001) (Table 2).

## Discussion

In this cohort, 29.1% of patients developed hypothyroidism following hemithyroidectomy, aligning with previously reported incidence rates ranging from 20% to 30%.^1^, ^4^, ^10^, ^14^ Preoperative TSH levels were significantly higher in patients who developed postoperative hypothyroidism, whereas thyroid lobe size, total thyroid volume, side of surgery, and resected lobe ratio did not significantly influence outcomes. This finding suggests that thyroid remnant size alone may not be a primary determinant of hypothyroidism risk, highlighting the predominance of biochemical and patient‐specific factors over anatomical variables.^9^, ^10^ This aligns with recent meta‐analytic data, including the systematic review and meta‐analysis by Cooper et al.^4^

Our results corroborate prior studies indicating that elevated preoperative TSH is a predictor of posthemithyroidectomy hypothyroidism.^14^, ^15^ Patients with baseline TSH levels above 2 mIU/L appear particularly vulnerable, supporting the need for close postoperative monitoring and early initiation of levothyroxine when clinically indicated. Interestingly, the temporal profile of hypothyroidism observed in our cohort aligns with previous reports: the majority of cases were identified within the first month postoperatively, while only a smaller fraction persisted at 6 months, suggesting potential compensatory adaptation of the remaining lobe.^1^, ^4^

Although patients with overt autoimmune thyroid disease were excluded, subclinical or seronegative autoimmune processes may still contribute to postoperative hypothyroidism. Previous studies have demonstrated lymphocytic infiltration even in the absence of detectable antibodies, suggesting that occult autoimmunity may impair compensatory capacity of the remaining lobe.^8^, ^9^, ^15^ This mechanism may partially explain why preoperative TSH, rather than gland size, predicts postoperative thyroid dysfunction and underscores the importance of considering latent autoimmune pathology when evaluating risk.

Additionally, systemic factors such as age, body mass index, diabetes, and overall metabolic reserve may influence postoperative outcomes. Emerging evidence suggests that markers of physiological reserve, such as skeletal muscle index (SMI), correlate with endocrine recovery following thyroid lobectomy, further indicating that hypothyroidism is a multifactorial process beyond local thyroid anatomy.^15^, ^16^

Unlike our study, a study conducted by Cherni et al, found that an unresected lobe volume <3.57 cm³ was significantly associated with the occurrence of hypothyroidism.^17^

Taken together, these findings emphasize a multifaceted risk assessment strategy for post‐hemithyroidectomy hypothyroidism, prioritizing preoperative biochemical evaluation, patient‐specific comorbidities, and potential latent autoimmune processes over thyroid volume or surgical side. This approach facilitates early identification of high‐risk patients, informs individualized follow‐up schedules, and guides timely initiation of thyroid hormone replacement therapy, ultimately optimizing clinical outcomes. Future prospective studies should aim to integrate biochemical, histopathologic, and systemic predictors to develop validated predictive models for postoperative hypothyroidism.^1^, ^9^, ^10^, ^15^, ^18^

### Limitations

A few limitations should be considered when interpreting the results of this study. First, the retrospective design inherently carries the risk of selection and information biases, as data collection relied on existing medical records, which may not capture all relevant variables. Second, the sample size was relatively modest, which may limit the statistical power to detect smaller associations, particularly for anatomical factors such as thyroid remnant volume or resected lobe ratio. Third, follow‐up was limited to 6 months postoperatively; longer‐term changes in thyroid function and late‐onset hypothyroidism could not be assessed.

The use of CT for volumetric assessment represents a limitation of this study. Although CT provides standardized and reproducible anatomical measurements, ultrasonography remains the clinical gold standard for thyroid volume evaluation; therefore, comparability with prior ultrasound‐based studies may be limited. In addition, the relatively small number of patients in certain Bethesda subcategories limited statistical power and precluded meaningful subgroup analyses based on cytology classification.

Despite these limitations, the study provides valuable data emphasizing the predictive role of preoperative TSH and patient‐specific factors over anatomical thyroid measures, and highlights areas for future prospective research.

## Further Research

Our study highlights that no relationship was found between thyroid gland volume and the development of hypothyroidism after hemithyroidectomy.

The role of autoimmune mechanisms—such as thyroid peroxidase (anti‐TPO) and thyroglobulin (anti‐Tg) antibodies—should be further explored, even in patients without known autoimmune thyroid disease. Surgical stress may unmask or accelerate latent autoimmune processes, thereby contributing to postoperative thyroid dysfunction.^19^, ^20^ For example, Walfish et al^21^ proposed that thyroid trauma and antigen release during surgery may trigger or reactivate autoimmune thyroid disease in predisposed individuals.

Future studies should also examine other systemic risk factors, including metabolic reserve, inflammatory markers, and perioperative stress responses, to determine whether they modulate the thyroid gland's ability to compensate after hemithyroidectomy. Identifying such predictors may enable the development of comprehensive risk stratification models that integrate biochemical, immunological, and systemic parameters, ultimately improving early detection and individualized management of hypothyroidism in this patient population.

## Conclusion

In conclusion, postoperative hypothyroidism following hemithyroidectomy appears to be more strongly associated with preoperative thyroid function than with anatomical thyroid volume. These findings emphasize the importance of biochemical assessment in preoperative risk stratification and postoperative follow‐up planning.

## Author Contributions

**Mayas Arraf**, MD, conceptualization, study design, data analysis, manuscript writing, and overall project coordination; **Alaa Safia**, MD, data collection and patient record management; **Adi Sharabi‐Nov**, PhD, MPH, statistical analysis and interpretation of results; **Moanes Awad**, MD, CT‐based thyroid volume measurements and imaging data analysis; **Yaniv Avraham**, BA, co‐writing and critical revision of the manuscript; **Ohad Ronen**, MD, critical review and supervision of the research process; **Shlomo Merchavy**, MD, study supervision, manuscript review, and final approval of the submitted version.

## Disclosures

### Competing interests

None.

### Funding source

None.
